# Preclinical Evaluation of Selene-Ethylenelacticamides in Tuberculosis: Effects Against Active, Dormant, and Resistant *Mycobacterium Tuberculosis* and In Vitro Toxicity Investigation

**DOI:** 10.3390/microorganisms13020396

**Published:** 2025-02-11

**Authors:** Natália Ferreira de Sousa, Maria Eugênia G. de Freitas, Maria Gabriella S. Sidrônio, Helivaldo Diógenes Souza, Alexia Czeczot, Marcia Perelló, Gabriela Fehn Fiss, Luciana Scotti, Demétrius A. M. de Araújo, José Maria Barbosa Filho, Cristiano V. Bizarro, Pablo Machado, Luiz Augusto Basso, Francisco Jaime B. Mendonça-Junior, Petrônio F. de Athayde Filho, Marcus T. Scotti, Valnês S. Rodrigues-Junior

**Affiliations:** 1 Graduate Program in Natural and Synthetic Bioactive Products, Federal University of Paraíba (UFPB), João Pessoa 58051-900, PB, Brazil; nataliafsousa@ltf.ufpb.br (N.F.d.S.); luciana.scotti@gmail.com (L.S.); barbosa.ufpb@gmail.com (J.M.B.F.); franciscojbmendonca@yahoo.com.br (F.J.B.M.-J.); mtscotti@gmail.com (M.T.S.); 2 Laboratory of Biotechnology in Microorganisms, Biotechnology Center, Federal University of Paraíba (UFPB), João Pessoa 58051-900, PB, Brazil; m.eugeniagouveia@gmail.com; 3 Graduate Program in Development and Technological Innovation in Medicines, Federal University of Paraíba (UFPB), João Pessoa 58051-900, PB, Brazil; gabriellassidronio@gmail.com; 4 Graduate Program in Chemistry, Federal University of Paraíba (UFPB), João Pessoa 58051-900, PB, Brazil; helivaldog3@gmail.com (H.D.S.); gffiss@gmail.com (G.F.F.); athayde-filho@quimica.ufpb.br (P.F.d.A.F.); 5 Programa de Pós-Graduação em Biologia Celular e Molecular, Escola de Ciências da Saúde e da Vida, Pontifícia Universidade Católica do Rio Grande do Sul (PUCRS) and Instituto Nacional de Ciência e Tecnologia em Tuberculose (INCT-TB)—Centro de Pesquisas em Biologia Molecular e Funcional (CPBMF), PUCRS, Porto Alegre 90619-900, RS, Brazil; alexiaczeczot@hotmail.com (A.C.); marcia.perello@pucrs.br (M.P.); cristiano.bizarro@pucrs.br (C.V.B.); pablo.machado@pucrs.br (P.M.); luiz.basso@pucrs.br (L.A.B.); 6 Graduate Program in Biotechnology (Renorbio), Department of Biotechnology, Biotechnology Center, Federal University of Paraíba (UFPB), João Pessoa 58051-900, PB, Brazil; demetrius@cbiotec.ufpb.br; 7 Programa de Pós-Graduação em Medicina e Ciências da Saúde, PUCRS and INCT-TB—CPBMF, PUCRS, Porto Alegre 90619-900, RS, Brazil; 8 Laboratory of Synthesis and Drug Delivery, Department of Biological Sciences, State University of Paraíba, João Pessoa 58071-160, PB, Brazil

**Keywords:** tuberculosis, drug development, selene-ethylenelacticamides, resistance, dormancy, toxicity

## Abstract

Selene-ethylenelacticamide derivatives have been suggested as promising scaffolds with leishmanicidal activity. In this work, we demonstrated, for the first time, the effectiveness of selene-ethylenelacticamide derivatives against mycobacteria. Firstly, selene-ethylenelacticamides inhibited the growth of laboratory strains of *Mycobacterium tuberculosis* with MIC values ranging from 10 to 20 µM. Importantly, three derivatives were active against two multi-drug-resistant clinical isolates of *M. tuberculosis* with MIC values similar to pan-sensitive strains. In addition, NC31 and NC34 displayed an improved activity compared to the group treated with isoniazid in the six-week nutrient-starved *M. tuberculosis* cultures. Moreover, in toxicity studies, NC34 did not significantly affect the viability of both Vero E6 and HepG2 cell lines. NC34 did not affect *Artemia salina* nauplii survival at concentrations lower than 100 µM. Importantly, NC34 displayed a synergistic effect when combined with rifampicin. Molecular docking simulations were used to evaluate *Mycobacterium tuberculosis* DprE1 and dihydrofolate reductase enzymes as putative targets of selene-ethylenelacticamides, mechanisms that could contribute to the antitubercular activity. Our findings reveal that NC34 may represent a hit for further drug optimization and for future preclinical development as a new anti-mycobacterial agent, especially in cases of resistant and/or dormant forms of tuberculosis.

## 1. Introduction

Tuberculosis (TB), caused by *Mycobacterium tuberculosis*, remains a major global health concern. According to the World Health Organization, TB was responsible for 1.25 million deaths in 2023 and will probably return to being the world’s leading cause of death from a single infectious agent, replacing COVID-19 [1]. Despite some progress in the pipeline for new drug candidates and regimens, there is still an urgent need for the development of new drugs to treat TB, especially those caused by the resistant forms of the bacillus. The emergence of multidrug-resistant- (MDR-) and extensively drug-resistant-TB (XDR-TB) has increased the need for developing innovative anti-TB therapies, as second-line drugs recommended for MDR- and XDR-TB are generally more toxic, more expensive, and less efficacious than the first-line drugs. Novel anti-TB drugs should ideally be effective against resistant strains, reduce the length of the treatment, require less frequent dosing, have minimal drug-drug interactions, and show reduced toxicity, improving patient adherence to the treatment [1,2,3].

Selenium compounds are chemical substances that contain the element selenium in their molecular structure [4]. Selenium is a chemical element belonging to the chalcogen group, similar to sulfur and tellurium, with unique properties that make it relevant in several areas, such as health, medicinal chemistry, biology, and technology [5,6]. Selenium compounds have attracted increasing interest in biomedical research due to their unique properties, including their ability to interact with essential biological molecules such as proteins, enzymes, and nucleic acids [7,8]. Among other therapeutic applications, these compounds show promise as antimicrobial agents due to their demonstrated efficacy against both Gram-positive and Gram-negative bacteria, including antibiotic-resistant strains such as methicillin-resistant *Staphylococcus aureus* [9,10,11].

The compounds in this work comprise 8 synthetic selene-ethylenelacticamide derivatives of ethylenelactic acid, obtained by De Sousa et al. [12] through organic synthesis guided by virtual screening studies using machine learning techniques combined with molecular docking simulations. These compounds showed a good spectrum of activity against *Leishmania* species, such as *Leishmania amazonensis*, *Leishmania infantum*, *Leishmania braziliensis*, and *Leishmania major* [12]. Their potential against mycobacteria remained unexplored. The main purpose of the present study was to investigate for the first time the potential of selene-ethylenelacticamides against actively growing, dormant, and resistant mycobacteria. In addition, our aim was to determine the safety of the more promising compounds in two different models: On cellular (Vero and HepG2) viability and on *Artemia salina* survival.

## 2. Materials and Methods

### 2.1. Drugs

**Figure 1:**
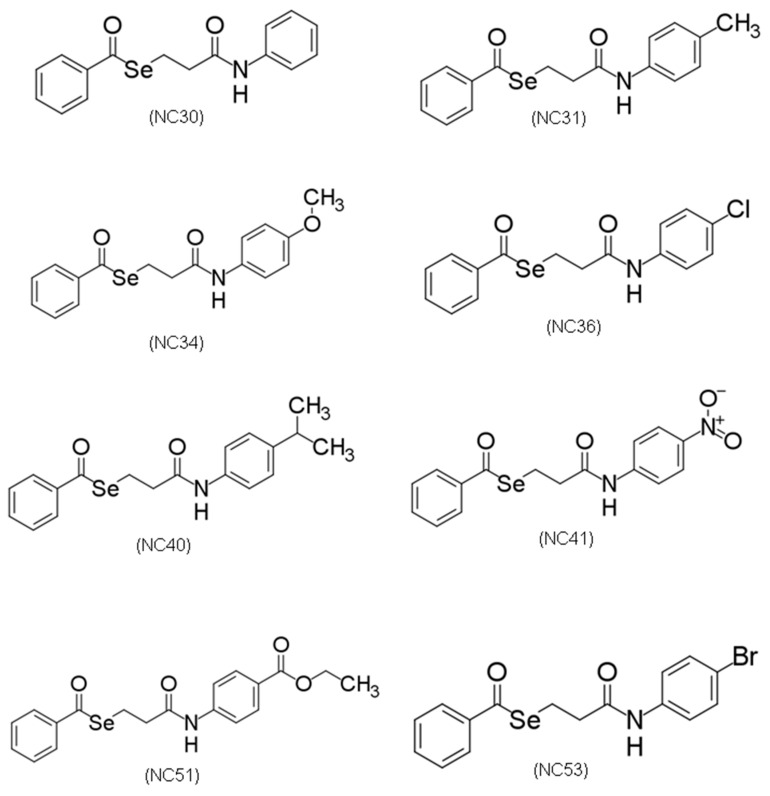
Chemical structure of the compounds under investigation: N-phenylbenzoselene-ethylenelactamide (NC30), N-(4-methylphenyl)benzoselene-ethylenelactamide (NC31), N-(4-methoxyphenyl)benzoselene-ethylenelactamide (NC34), N-(4-chlorophenyl)benzoselene-ethylenelactamide (NC36), N-(4-isopropylphenyl)benzoselene-ethylenelactamide (NC40), N-(4-nitrophenyl)benzoselene-ethylenelactamide (NC41), Ethyl (4-benzoselene-ethylenelacticamide) benzoate (NC51), N-(4-bromophenyl)benzoselene-ethylenelactamide (NC53).

Isoniazid, rifampicin, moxifloxacin, and ethambutol were purchased from Sigma-Aldrich. Selene-ethylenelacticamides were synthesized as previously described [12]. The compounds of the series under study are shown in Figure 1.

### 2.2. Screening for Anti-Mycobacterial Potential

The anti-mycobacterial activity was determined by using the resazurin reduction microplate assay (REMA) as a growth indicator [13]. *M. tuberculosis* H37Ra suspensions were grown in Middlebrook 7H9, with 10% OADC (oleic acid, albumin, dextrose, and catalase; Becton Dickinson), 0.2% glycerol, and 0.05% Tween-80. Briefly, test compounds were first solubilized in dimethyl sulfoxide (DMSO) at a concentration of 8 mM and then diluted in Middlebrook 7H9 + 10% OADC broth to reach a concentration of 160 µM. Serial two-fold dilutions were performed in 96-well U-bottom polystyrene microplates at concentration ranges of 160–1.25 µM for all compounds. The DMSO concentration was maintained at 2.5% in all experimental groups. Mycobacterial suspensions were diluted in 7H9 medium at an optical density (OD_595nm_, WPA UVA 1101 Biotech Photometer—Gemini BV) of 0.006, and 100 µL were added to each well. Following incubation at 37 °C for 7 days, 30 µL of a sterile resazurin solution (0.02%) was added to the plates, and the color (blue or pink) of each well was visually read after 48 h [14]. Minimum inhibitory concentrations (MICs) were considered the lowest compound concentration that prevented a color change from blue (resazurin) to pink (resorufin). Isoniazid and rifampicin were used as positive control drugs.

### 2.3. Determination of MIC Against Virulent and Resistant M. Tuberculosis

Drugs that exhibited bacterial growth inhibition at MICs of 10 or 20 µM were chosen for additional testing against the drug-sensitive H37Rv strain and MDR clinical isolates of *M. tuberculosis*, PT-2, and PT-20. MDR PT-2 harbors genetic changes in *inh*A (S94A) and *rpo*B (S531L) genes and in the promoter sequence of *inh*A, [C(-15)T]. MDR PT-20 clinical isolates carry genetic changes in *kat*G (S315T) and *rpo*B (S531L) genes. The MICs were determined using the REMA method. Mycobacterial suspension cultivation, assay procedures, and result analyses were performed as previously described in Section 2.2, screening for anti-mycobacterial potential [13,14].

### 2.4. Toxic Effect in the Nutrient Starvation Model

Mycobacterial nutrient-starved suspensions were prepared as previously described [15]. Briefly, *M. tuberculosis* was cultured in Middlebrook 7H9, plus 10% OADC enrichment, 0.2% glycerol, and 0.05% Tween-80. After two to three weeks of cultivation, suspensions were centrifuged and washed twice with sterile phosphate-buffered saline (PBS). The cellular sample was mixed with PBS buffer, placed in airtight containers, and kept at a temperature of 37 °C for a period of six weeks [15]. The six-week-starved and actively growing suspension of *M. tuberculosis* was then incubated for 7 days with NC31 and NC34 at 10 µM, with concentrations corresponding to the MIC for NC31 and to half of the MIC value for NC34. Isoniazid was used as a control at 10 µM. DMSO (2.5%), the vehicle, was included in all experimental groups. Aliquots were successively diluted and inoculated onto Middlebrook 7H10 Agar (Difco) with 10% OADC. Mycobacterial colony forming units (CFU) were enumerated after incubation of plates for three weeks at 37 °C. This test was performed in quadruplicate, and the results are expressed as the log mean numbers (± standard error of the mean) of CFU per well.

### 2.5. Cytotoxicity Investigation

The lead compounds NC31 and NC34 were selected for toxicity investigations. Cellular viability determination after incubation with the test compound was performed as described by Meerloo et al., 2011 [16]. African green monkey kidney (Vero) and human hepatoma (HepG2) cells, obtained from Cell Bank of Rio de Janeiro, were cultured in DMEM media (Dulbecco’s Modified Eagle Medium) supplemented with 10% inactivated fetal bovine serum (FBS) and 1% antibiotics (penicillin-streptomycin). The cells were maintained in culture flasks at 37 °C in a humidified atmosphere with 5% CO2. Cells were seeded at 5 × 10^3^ and incubated overnight to adhere. The following day, the cellular samples underwent treatment with solutions of NC31 and NC34, resulting in concentrations ranging from 12.5 to 200 µM (DMSO 1%, *v/v*). After 72 h at 37 °C under 5% of CO_2_, all wells received MTT solution (0.5 mg/mL) for 3 h. The formazan crystals were dried overnight at room temperature and dissolved in DMSO. The optical density was recorded in 570 nm (Absorbance microplate reader EL800, BioTek, Winooski, VT, USA). The percentages of cell viability for treated groups were reported considering the control wells (DMSO 1–treated) as 100% of cell viability. Data were expressed as the mean of cell viability ± the standard error of the mean of three to four independent experiments performed in triplicates.

### 2.6. Artemia Salina Toxicity Evaluation

*Artemia salina* survival determination after incubation with the test compounds NC31 and NC34 was performed as described by Magalhães et al., 2025 [14]. *Artemia salina* (brine shrimp) Dormant eggs were obtained from a local fish supply store and incubated (0.5 g cysts/L) in modified artificial seawater (sea salt 35 g/L) supplemented with 6 mg/L of dried yeast. Aeration was provided by a line extending to the bottom of the hatching device from an aquarium air pump. After 2 days of incubation (in a 23–25 °C room), larvae were gathered using a glass dropper after drawing the organisms toward a single edge of the incubation container by illuminating that area. Lead drug solutions were serially 2-fold diluted and placed in 96-well microplates in quadruplicates in 100 µL of 0.9% NaCl solution with 6 mg/L of dried yeast. The final concentration of DMSO was 2.5% for all treatment groups, including for the control wells. Into each well, we pipetted 100 µL of solution containing between 8 and 12 nauplii, then placed the plate in an incubator at 23–25 °C for 24 h. The quantification of alive versus immotile nauplii for all experimental groups was then enumerated by examining them under a microscope. Nauplius was considered to be immobile or dead if it did not move for 10 s in the presence of light. The percentages of nauplii viability for each drug concentration evaluated were determined, considering the percentage of survival from the control group (2.5% DMSO) as 100%.

### 2.7. Statistical Analysis

Data from *Artemia salina* survival, cytotoxicity, and effects in the nutrient starvation model were evaluated by one-way analysis of variance, followed by Bonferroni’s post-test, using GraphPad Prism 8.0 (San Diego, CA, USA). Differences were considered significant at the 95% level of confidence.

### 2.8. Compound Interactions

Interactions of NC34 with moxifloxacin, ethambutol, rifampicin, and isoniazid were investigated using a checkerboard assay in a two-drug association scheme using the REMA colorimetric method as a growth indicator, as previously reported [13]. Briefly, drugs were diluted in 7H9 + OADC to obtain concentration ranges in microplates of 0.94–0.06 µM for isoniazid, 0.06–0.004 µM for rifampicin, 16–1 µM for ethambutol, 0.4–0.025 µM for moxifloxacin, and 20–1.25 µM for NC34. NC34 was diluted vertically (rows A to E), while combined drugs were added horizontally (columns 1 to 5) (Supplementary Material). The concentration of DMSO in all wells was maintained at 2.5%. The *M. tuberculosis* inoculum, the incubation conditions of the microplates, and the readout of results were carried out as described before for MIC determination [13,14]. The fractional inhibitory concentration index (FICI) has been determined for each combination. Results lower than 0.5 would suggest that the drugs work together beneficially (synergy), values from 0.5 to 4 would suggest that the compounds work independently (indifference), and values higher than 4 would indicate that the drugs interfere with each other (antagonism) [17].

### 2.9. Molecular Docking

Molecular docking simulation was used to evaluate the mechanism of synthetic compounds of selene-ethylenelacticamides that contribute to their antitubercular effect through the surface layer of these targets: *Mycobacterium tuberculosis* DprE1 (Decaprenylphosphoryl-β-D-ribose 2′-epimerase) in complex with CT319 (PDB: 4FDO) [18], with a resolution of 2.40 Å and obtained by the X-ray diffraction method, and dihydrofolate reductase complexed with beta-NADPH and 3′-(3-(2,4-diamino-6-ethylpyrimidin-5-yl)prop-2-yn-1-yl)-4′-methoxy-[1,1′-biphenyl]-4-carboxylic acid (UCP1106) (PDB: 5JA3) [19], with a resolution of 1.81 Å and obtained using the X-ray diffraction method.

Prior to choosing the targets under study, a literature search was carried out in order to select targets crucially related to survival, taking into account mechanisms related to replication and energy metabolism of the microorganism under study. The active site was demarcated according to the region delimited by the co-crystallized ligand of the enzymes under study, corresponding to 3-nitro-N-[(1R)-1-phenylethyl]-5-(trifluoromethyl)benzamide for the enzyme DprE1 (PDB: 4FDO) and the compound 4-[3-[3-[2,4-bis(azanyl)-6-ethyl-pyrimidin-5-yl]prop-2-ynyl]-4-methoxy-phenyl]benzoic acid for the enzyme dihydrofolate reductase (PDB: 5JA3).

The synthetic compounds derived from selenium were designed in the Marvin Sketch 24.1.3 ChemAxon software (https://docs.chemaxon.com/ accessed on 22 November 2024) and were prepared with the addition of hydrogen atoms to the chemical structure and subsequent conversion into the third 3D dimension.

To evaluate the occurrence of structural deviations between the PDB ligand and its most stable pose, redocking was performed prior to the molecular docking calculations, which was evaluated by the RMSD (Root Mean Square Deviation) metric, which ensures correct positioning of the poses within a value of up to 2.0 Å [20]. The docking and redocking procedures were performed using the Molegro Virtual Docker 6.0.1 software, and the enzymes and compounds under study were prepared according to parameters predefined in the software.

In the coupling procedure (linker–enzyme), a grid of 15 Å radius and a resolution of 0.30 was utilized with 30 runs. This grid encompassed the binding site, as defined by a known ligand for each enzyme. A model was generated to perform and evaluate the fit with expected characteristics between the ligand and the enzyme, using the MOLDOCK Score (GRID) algorithm with the scoring function and search algorithm corresponding to Moldock [21,22]. The MolDock scoring function enhances these scoring functions with a new hydrogen bonding term and new charge schemes. The docking scoring function, Escore, is defined by the following energy terms:

Escore = Einter + Eintra

The visualization of the established interactions was performed in the Discovery Studio Visualizer program, Biovia, 2021 v21 1.0 (https://www.3dsbiovia.com/ accessed on 22 November 2024).

## 3. Results

### 3.1. Selene-Ethylenelacticamides Are Active Against M. Tuberculosis Laboratory Strains

We first determined the antimycobacterial potential for NC30, NC31, NC34, NC36, NC40, NC41, NC51, and NC53 in *Mycobacterium tuberculosis* H37Ra. The MIC for NC36, NC51, and NC53 was found to be 40 µM, and for NC41, 80 µM. As presented in Table 1, NC30, NC31, NC34, and NC40 suppressed the growth of *M. tuberculosis* H37Ra with MIC values of 10 or 20 µM. The MICs found for the standard therapeutic agents utilized in these assays, isoniazid and rifampicin, were 1.9 and 0.03 µM, respectively.

NC30, NC31, NC34, and NC40 were consequently chosen for additional evaluation using the virulent laboratory H37Rv strain of *M. tuberculosis*. NC31 and NC34 were the more potent molecules, with MICs of 10 µM. MICs for NC30 and NC40 resulted in 20 µM and 40 µM, respectively (Table 1). The values reported here were observed in three independent experiments or were the highest values found among three independent tests.

### 3.2. Selene-Ethylenelacticamides Are Active Against MDR Clinical Isolates of M. Tuberculosis

As a related effort, we analyzed the impacts of NC30, NC31, NC34, and NC40 against drug-resistant clinical isolates of *M. tuberculosis*. Two MDR clinical isolates (PT-2 and PT-20), genetically defined earlier [23], were evaluated. The MDR clinical isolates harbor the same genetic change (S531L) in the *rpo*B gene, responsible for generating resistance to rifampicin. PT-2 carries a mutation [C(-15)T] in the promoter sequence of the *inh*A (Rv1484) gene and also carries a genetic change (S94A) in the *inh*A gene, which encodes the target for isoniazid. PT-20 carries the mutation (S315T) in the *kat*G (Rv1908c) gene, which represents the predominant genetic alteration identified in isoniazid-resistant strains. Of note, NC31, NC34, and NC40 affected the viability of MDR strains of *M. tuberculosis* with MIC results of 20 µM (Table 1). These results indicate that NC31, NC34, and NC40 could overcome principal resistance mechanisms found in resistant clinical isolates of *M. tuberculosis*, such as genetic modifications in either *kat*G or *rpo*B genes.

### 3.3. Selene-Ethylenelacticamides Lowered Mycobacterial CFU Loads in the Nutrient Starvation Model

**Figure 2:**
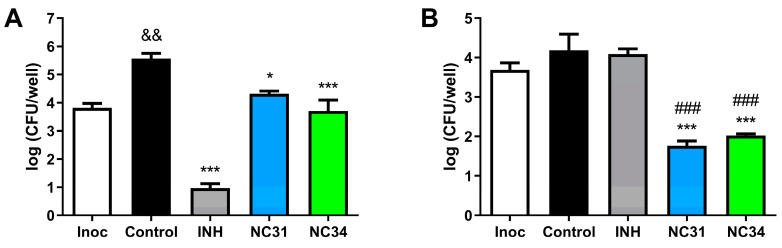
Toxic effect of NC31 and NC34 against non-starved log phase (**A**) and nutrient-starved (**B**) *Mycobacterium tuberculosis*. The control group was incubated with the vehicle, 7H9 medium, and 2.5% DMSO. INH, isoniazid; Inoc, representing the bacterial inoculum in the day treatments were added. && *p* < 0.01 compared to Inoc; *** *p* < 0.001, * *p* < 0.05 compared to control group; ### *p* < 0.001 compared to isoniazid-treated group.

Considering the highest potency against laboratory strains and clinical isolates of *M. tuberculosis,* we have chosen NC31 and NC34 for further evaluation and then determined their activity in a nutrient starvation model for *M. tuberculosis*. The actively growing suspensions of *M. tuberculosis* incubated with NC31, NC34, or isoniazid exhibited markedly diminished CFU counts (Figure 2A) when compared to the untreated group (*p* < 0.001 for treatments with isoniazid or NC34 and *p* < 0.05 for treatment with NC31). Decreases in bacterial loads ranged from 1.3 to 4.6 log_10_ for treatment wells. Nevertheless, in nutrient-starved *M. tuberculosis* cultures, incubation with isoniazid caused a decrease of 0.09 log_10_ in the CFU/well loads (Figure 2B). Of importance, treatment with NC31 decreased bacterial loads in relation to the control group (*p* < 0.001) and resulted in an enhanced activity over to the group incubated with isoniazid (*p* < 0.001) in the dormancy experiment. Likewise, marked contrasts of CFU counts were found comparing NC34-treated wells versus the untreated control (*p* < 0.001) and isoniazid groups (*p* < 0.001) (Figure 2B).

### 3.4. Cytotoxicity Evaluation of NC31 and NC34 on Vero E6 and HepG2 Cell Viabilities

**Figure 3:**
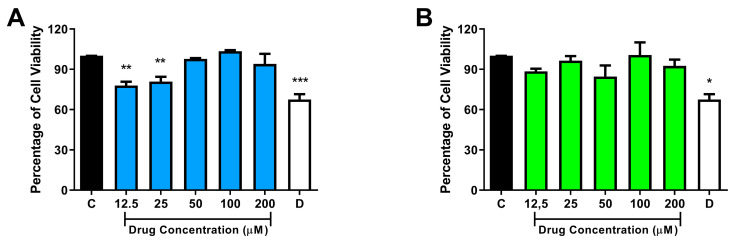
Cytotoxicity investigation. Data of cytotoxic effects of test compounds NC31 (**A**) and NC34 (**B**) on Vero E6 cells. C, Control: 1% DMSO-treated wells, considered as 100% cell viability; D: 10% DMSO. Data were expressed as the mean of cell viability ± standard error of the mean of three to four independent experiments performed in triplicate. *** *p* < 0.001, ** *p* < 0.01, * *p* < 0.05, compared to the corresponding control group.

**Figure 4:**
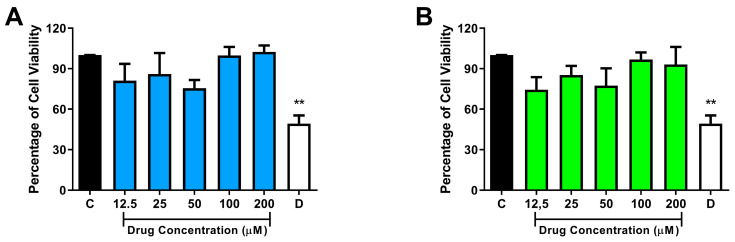
Cytotoxicity investigation. Data of cytotoxic effects of test compounds NC31 (**A**) and NC34 (**B**) on HepG2 cells. C, Control: 1% DMSO-treated wells, considered as 100% cell viability; D: 10% DMSO. Data were expressed as the mean of cell viability ± standard error of the mean of three to four independent experiments performed in triplicate. ** *p* < 0.01, compared to the corresponding control group.

We have investigated the possible in vitro cytotoxic effects of compounds NC31 and NC34 using the MTT assay. African green monkey kidney (Vero) and HepG2 hepatic cells were used in these experiments. Cellular viability was evaluated after the exposition of the cell lineages with the compounds (NC31 and NC34) for 72 h. The results were expressed as a percentage of cell viability, considering the 1% DMSO-treated control wells as 100% of cell viability. In toxicity experiments, NC31 demonstrated significant cytotoxicity against Vero cells at two distinct concentrations, 12.5 and 50 µM, over a 72 h period (Figure 3A). The difference was not statistically significant after comparing the means of HepG2 cell viability of the NC31-treated groups with the corresponding 1% DMSO-treated control (Figure 4A). Of note, the in vitro incubation of the compound NC34, at concentrations ranging from 12.5 to 200 µM, did not significantly affect cell viability of these two eukaryotic cell lines (Figure 3B and Figure 4B).

### 3.5. Toxicity Evaluation of NC31 and NC34 on Artemia Salina Survival

**Figure 5:**
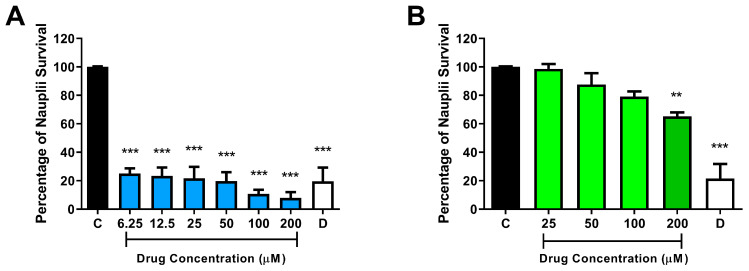
Effects of test compounds NC31 (**A**) and NC34 (**B**) on *Artemia salina* survival after 24 h of exposure. C: Control, 2.5% DMSO vehicle, set as 100% of *Artemia salina* survival; D: 10% DMSO. Data were expressed as the mean of nauplii survival ± standard error of the mean of two independent experiments performed in quadruplicate. ** *p* < 0.01, *** *p* < 0.001 compared to the corresponding control (C) group.

NC31 and NC34 underwent toxicity investigation using *Artemia salina* (brine shrimp) bioassay. Initially, the preliminary assessment tested varying concentrations of 25 to 200 µM demonstrated that NC34 (at 25 to 100 µM) did not significantly affect nauplii survival rates over a 24 h period (Figure 5B). The group treated with the highest NC34 concentration, 200 µM, resulted in a nauplii survival mean of 65% (± 3), suggesting that the LC_50_ value is higher than 200 µM for NC34. An experimental group received 10% DMSO as a mortality control. In contrast, NC31 significantly reduced nauplii survival for all tested concentrations, from 6.25 to 200 µM (Figure 5A).

### 3.6. NC34 Resulted in a Synergistic Interaction Plus Rifampicin

The outcomes of the two-drug interactions of NC34 with therapeutic agents used in clinical practice against TB moxifloxacin, ethambutol, rifampicin, and isoniazid were evaluated. As shown in Table 2, NC34 showed an indifferent interaction when combined with isoniazid, ethambutol, and moxifloxacin (FICI results from 1 to 2.5). Of importance, NC34 displayed a synergistic interaction when combined with rifampicin (FICI results of 0.38 in three independent tests). Both the MIC of NC34 in the presence of rifampicin and the MIC of rifampicin in the presence of NC34 were enhanced from four- to eight-fold in all three experiments (Table 2). Importantly, no two-drug combination evaluated in this work presented antagonism.

### 3.7. Molecular Docking Studies

Synthetic selenium derivatives belonging to the selene-ethylenelacticamide class were subjected to molecular docking simulations with the following proteins: DprE1 (PDB: 4FDO) and dihydrofolate reductase (PDB: 5JA3), and the results were generated according to the energy score of the MolDock Score algorithm and lower energies indicated a greater affinity. The validation of the targets under study by Redocking was performed prior to the molecular docking simulations by evaluating the RMSD (Root Mean Square Deviation) values. The RMSD values for the proteins under study corresponded to 0.2379 for the fluoromethylbezamide ligand of the DprE1 protein (PDB: 4FDO) and 0.2151 for the benzoic acid-derived ligand of the dihydrofolate reductase protein (PDB: 5JA3). Thus, it can be concluded that the results validate the targets under study and confirm the correct reproduction of the poses of the ligands under study by the Molegro Virtual Docker (MVD) software.

The molecular docking results of the compounds under study and their respective ligands for the targets under study are available in Table 3.

**Figure 6:**
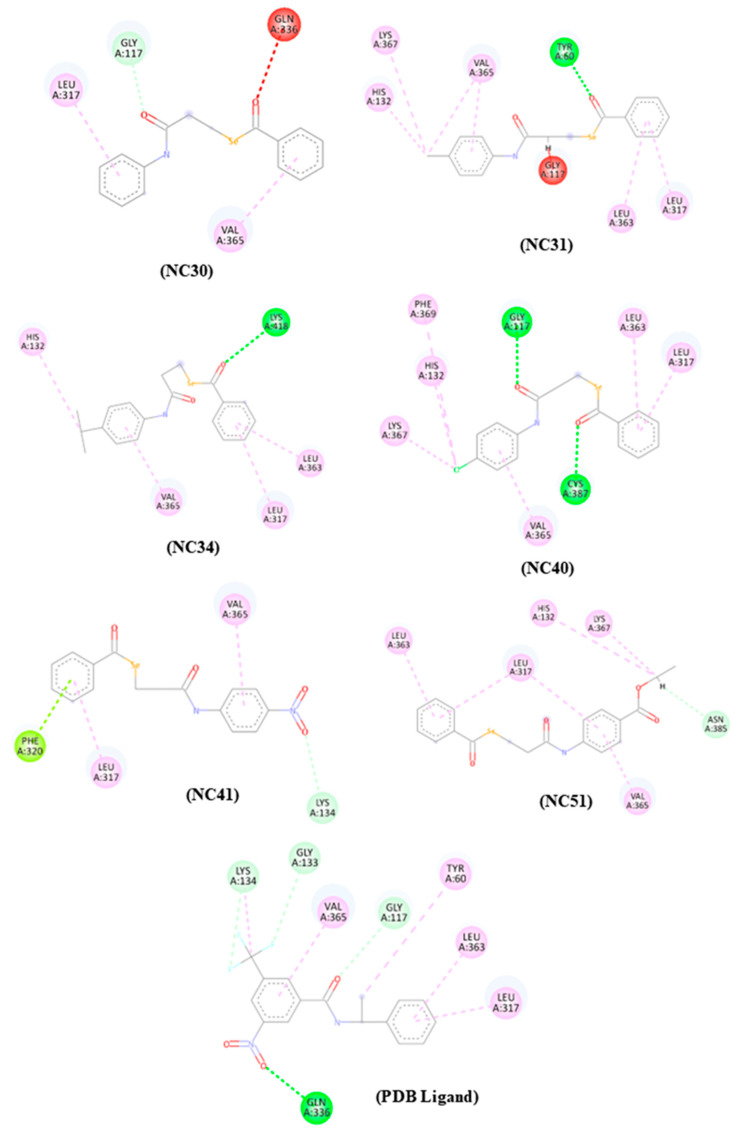
Molecular interaction maps of compounds NC30, NC31, NC34, NC40, NC41, NC51, and PDB ligand trifluoromethylbenzamide derivative. Interactions: Light pink (alkyl and Pi-alkyl), light green (Carbon hydrogen bond), dark green (conventional hydrogen interaction), red (unfavorable interaction), and lime green (Pi-lone pair interaction). Residues: Leu (Leucine), Gly (Glycine), Gln (Gkutamine), Val (Valine), His (Histidine), Lys (Lysine), Tyr (Tyrosine), Phe (Phenylalanine), Cys (Cysteine), and Asn (Asparagine).

According to the results observed in Table 3, it is possible to notice that the selene-ethylenelacticamide derivatives presented negative affinity score values for all targets, indicating that interaction with the enzymes under study occurred. Furthermore, it was observed that four compounds under study presented lower and/or very close affinity score values when compared to the PDB ligand of the target DprE1 (PDB: 4FDO). These compounds are NC51 (−95,193), NC41 (−86,800), NC40 (−87,517), and NC31 (−81,121). It is important to highlight that compounds NC31 and NC40 showed affinity to the target under study, as well as favorable results in the biological test performed. Although compounds NC30 (−73.214) and NC34 (−75.389) did not present lower binding energy values when compared to the PDB ligand, it was observed that these compounds presented negative score values, as well as values close to the values demonstrated by the PDB ligand with a difference of only −13.758 for compound NC30 and −11.583 for compound NC34. Another important point observed corresponded to the fact that the compounds with the highest affinity were substituted with the ethyl ester (NC51), isopropyl (NC40), nitro (NC41), methyl (NC31), methoxy (NC34), and unsubstituted benzene (NC30) groups. Among these, the unsubstituted benzene compound (NC30) showed the lowest score, likely due to its fewer heavy atoms. This atomic composition notably affected the scoring of compound NC51, as the MolDock Score algorithm calculates binding affinity based on a weighted sum of interaction and deformation energies. The algorithm incorporates penalties and adjustments for solvation, considering various energy contributions that describe ligand-receptor interactions and ligand internal conformation [21]. For the enzyme dihydrofolate reductase, molecular docking analysis revealed that the PDB ligand exhibited the highest stability, with a binding score of −155.333. Figure 6 illustrates the molecular interaction maps between the compounds under study with the enzyme DprE1 (PDB: 4FDO). The analysis revealed multiple types of interactions, including hydrogen bonds (depicted by green dashed lines) and hydrophobic interactions (shown in pink dashed lines). Of note, these interactions demonstrate significant binding between the aromatic and ketone groups of the studied compounds and key residues within the enzyme.

The molecular interaction maps (Figure 6) of the compounds under study were observed important residues that are essential for enzyme inhibition, such as residue Lys 134 (Compound 7—NC41), Gly117 (Compound 1—NC30, Compound 2—NC31 and Compound 4—NC40), Lys367 (Compound 2—NC31, Compound 4—NC40 and Compound 8—NC51), Tyr60 (Compound 2—NC31), Asn385 (Compound 8—NC51), Cys387 (Compound 4—NC40), His132 (Compound 2—NC31, Compound 3—NC34, Compound 4—NC40 and Compound 8—NC51), Gln336 (Compound 1—NC30), Leu363 (Compound 2—NC31, Compound 3—NC34, Compound 4—NC40, and Compound 8—NC51) [18]. It is worth mentioning that the Cys387 residue is located in the active site of the DprE1 enzyme (PDB: 4FDO) and establishes a covalent interaction, also establishing interaction with the Flavin Adenine Dinucleotide (FAD) cofactor since this is located in the active site [18]. In addition to this residue, it is observed that the active site of the enzyme, together with the site occupied by the FAD cofactor, presents other residues that correspond to Lys134, Gly117, Tyr60, Asn385, Cys367, His132, Gln336, and Leu363, as these residues comprise the active site region, and thus represent an important contact point for inhibitors [18]. Modification or interaction with these residues, especially with the Cys387 residue, can alter the activity of the protein, impacting processes fundamental for the survival of *M. tuberculosis* [18]. Since the enzyme DprE1 (PDB: 4FDO) is one of the main targets involved in the biosynthesis of arabinogalactan, an essential component of the cell wall of *M. tuberculosis*, it plays a crucial role in the epimerization of decaprenylphosphate-ribose (DPR) into decaprenylphosphate-arabinose (DPA), which is a necessary precursor for the construction of the mycobacterial cell wall [24]. Thus, inhibition of the enzyme DprE1 interrupts the biosynthesis of arabinogalactan, an essential component of the cell wall of *M. tuberculosis* [25]. Without arabinogalactan, the cell wall structure is destabilized, leading to the loss of cellular integrity and, consequently, the death of the mycobacteria [26].

Furthermore, similar interactions of the compounds under study with the PDB ligand corresponding to Leu317 and Val365 were observed, referring to alkyl-type hydrophobic interactions, indicating that the compounds may occupy the same site.

## 4. Discussion

In this work, we determined that NC30, NC31, NC34, and NC40 inhibited the growth of *M. tuberculosis* H37Ra, H37Rv, and MDR *M. tuberculosis* clinical isolates. While no previous studies had directly investigated the potential of selene-ethylenelacticamides against *Mycobacterium* spp., several related studies have explored selenium-containing compounds. Ribeiro et al. demonstrated that a new series of selenium-containing naphthoquinones was active against *M. tuberculosis* H37Rv and MDR clinical isolates [27]. Estevez et al. established the mycobactericidal capacity of selenium nanoparticles against both *M. smegmatis* and *M. tuberculosis* [28]. Additionally, Chitra et al. observed that a series of mono and bis-1,2,3-selenadiazole derivatives showed in vitro activity against *M. tuberculosis* H37Rv [29].

The results of this study indicate that selene-ethylenelacticamide derivatives can overcome the major mechanisms of resistance found in drug-resistant clinical isolates of *M. tuberculosis*, supporting further investigation of these compounds in in vivo preclinical tests for developing new antituberculosis agents. Furthermore, the absence of resistance observed in the two clinical isolates suggests these compounds act through mechanisms distinct from first-line drugs such as isoniazid and rifampicin. The effects of combining NC34 with clinically used anti-TB drugs support this hypothesis, as no antagonistic interactions were observed in our experiments. Notably, NC34 displayed synergistic effects when combined with rifampicin. To better understand the precise mechanisms by which these compounds inhibit mycobacterial growth, further studies examining structure-activity relationships are necessary.

The development of dormancy-based tests with *M. tuberculosis* enables the evaluation of bacilli exhibiting slow in vitro growth, reduced metabolic activity, and resistance to antimycobacterial agents, which aids in understanding TB pathogenesis and testing novel therapeutic regimens [30]. Models using nutrient deprivation have demonstrated that *M. tuberculosis* can survive for extended periods in a non-growing state, and this condition has been shown to induce *M. tuberculosis*-antibiotic tolerance to isoniazid and rifampin in vitro. Evidence also suggests that persistent bacilli in lung lesions experience nutrient deprivation [15].

In this study, we demonstrated that NC31 and NC34 derivates effectively reduced CFU counts of dormant bacteria. While no previous studies have investigated selenium derivatives against dormant mycobacteria, our results indicate that treatment with NC31 and NC34 showed improved potency compared to isoniazid against the nutrient-starved bacteria. The decreased efficacy of isoniazid observed in the six-week nutrient-starved *M. tuberculosis* model aligns with earlier observations [15,31]. These observations suggest that NC31 and NC34 demonstrate promising activity in the nutrient-starved *M. tuberculosis* model.

The development of toxicity tests is essential for drug development. In vitro models represent valuable tools for evaluating preliminary toxic effects in specific cell types, offering a rapid and cost-effective method for screening cellular responses [32]. The monkey kidney cell line Vero E6 has been widely used in toxicological research. In the present work, we observed that NC31 significantly reduced Vero cell viability at only two concentrations (12.5 and 50 µM) over a 72 h period. Notably, the in vitro incubation of compound NC34 at concentrations ranging from 12.5 to 200 µM did not significantly affect cell viability of the Vero E6 cell line.

No previous studies have investigated the potential of selene-ethylenelacticamides on Vero E6 cells. However, studies examining the cytotoxic potential of other selenium derivatives against Vero E6 have been reported. Tucci et al. demonstrated, using a methylene blue assay, that chalcogen-zidovudine derivatives containing selenium presented IC50 values ≥ 100 µM for Vero E6 models [33]. Majeed et al. showed that selenium-enriched garlic powder at a concentration of 25 μg/mL presented with 89% cell viability, which was established as the highest permissible non-cytotoxic concentration over a 24 h period [34]. Importantly, our results suggest that NC34 may be a suitable candidate for further efficacy and safety testing in murine models.

HepG2 cells, a tumor cell line derived from the human liver, demonstrate, in comparison with normal hepatocytes, enhanced capabilities in maintaining and amplifying alterations in systems related to the metabolism of endogenous and exogenous substances. This attribute makes HepG2 cells a valuable model for studying the metabolism of new drug candidates [35]. In this work, we used the HepG2 cell line to predict the hepatic toxicity of NC31 and NC34. Our results showed that NC31 and NC34 did not affect the viability of the HepG2 cell line at any of the tested concentrations. This study represents the first evaluation of selene-ethylenelacticamide derivatives’ effects on the HepG2 cell line. We then estimated that the IC_50_ value for NC34 is higher than 200 µM for both eukaryotic cell lines tested. The selectivity index is calculated to be higher than 10, based on the minimum inhibitory concentration of 20 µM.

Wang et al. studied the protective mechanism of selenocystine against methylmercury cytotoxicity in HepG2 cells. Selenomethionine showed no toxicity at concentrations ranging from 0 to 150 μmolL^−1^, while selenocystine and methyl-Se-cysteine exhibited mild toxicity at concentrations exceeding 60 μmolL^−1^ [36]. In their study, Zeebaree et al. reported that HepG2 cells treated with biologically fabricated selenium nanoparticles showed an IC_50_ of 3.98 μg/mL [37]. The variations in these results may be attributed to the different organic groups present in the molecular structures of these compounds. Taken together, our data suggest that NC31 and NC34 do not exhibit hepatotoxicity and are promising candidates for further toxicological studies.

The use of *A. salina* in acute toxicity assays offers significant potential due to its simplicity, low cost, and reproducibility [38]. In the present study, we demonstrated that NC34 did not significantly affect nauplii survival rates over a 24 h period at lower concentrations; however, the group treated with the highest concentration (200 µM) of NC34 showed reduced nauplii survival. Our study is the first to demonstrate selene-ethylenelacticamide toxicity in this model system. Our findings reveal that NC34 may represent a promising lead compound for further drug optimization and future preclinical development as a new anti-mycobacterial agent with a favorable toxicity profile, particularly for treating MDR and quiescent forms of TB.

## 5. Conclusions

In this study, we present, for the first time, the activity of selene-ethylenelacticamide derivatives against both drug-sensitive and drug-resistant mycobacteria. The results indicate that these derivatives have the potential to overcome the main resistance mechanisms found in MDR clinical isolates of *M. tuberculosis*. However, the development of assays aimed at identifying the structure-activity relationships is necessary to elucidate the pathways through which these compounds affect the viability of mycobacteria. Additionally, the findings suggest that NC31 and NC34 derivatives impact the viability of dormant bacteria, allowing us to propose that these compounds may represent significant advancements in drug optimization of new antimycobacterial agents. Combination tests of selene-ethylenelacticamide derivatives with drugs used in the treatment of TB showed that the NC34 derivative exhibits a synergistic effect when combined with rifampicin, suggesting its great potential for co-administration with clinically available drugs, especially in the case of drug-resistant forms of TB. In summary, these results play an important role in advancing our understanding of the antimycobacterial potential of selene-ethylenelacticamide derivatives as candidates for TB treatment. However, further testing is essential to confirm the findings of this study.

## 6. Limitations

Although this study revealed the anti-mycobacterial potential of selene-ethylenelacticamide derivatives, some important limitations should be considered. There are still no pharmacokinetic (PK) data for the lead compounds, so we still do not know whether it reaches the 10–20 µM concentrations when administered to rodents. Possibly, PK/pharmacodynamic studies using hollow fiber models can help predict the early PK profile for these drug candidates. Furthermore, the experiments were performed exclusively in in vitro models, limiting the extrapolation of the results to in vivo systems. In this regard, conducting experiments in murine models will provide a more robust validation of the results while also enabling the analysis of the efficacy in the mouse model of TB, early bactericidal activity, and potential in vivo toxic effects of the lead molecules.

## 7. Patents

The compounds of this work were patented as an unprecedented substance for tuberculosis. The process was submitted to the National Institute of Industrial Property (INPI) through the INOVA/UFPB agency, having been accepted on December 22, 2021, under case number BR 10 2021 026090 4 and petition 870210119497.

## Figures and Tables

**Table 1:** Minimum inhibitory concentrations (MIC, in µM) of selene-ethylenelacticamide drugs against MDR *M. tuberculosis* clinical isolates and *M. tuberculosis* laboratory strains.

Drug	MIC (µM) *^a^*			
	*M. tuberculosis* H37Ra	*M. tuberculosis* H37Rv	PT-2	PT-20
NC30	20	20	40	40
NC31	10	10	20	20
NC34	20	10	20	20
NC40	20	40	20	20
Isoniazid	1.9	2.3	292	292
Rifampicin	0.03	0.05	>80	>80

*^a^* The values shown in this work were observed in two independent experiments or were the highest values found among three independent tests.

**Table 2:** NC34 drug candidate and rifampicin had a synergistic interaction against *M. tuberculosis*.

Drug Combination	MIC (µM) *^a^*		FICI *^b^*	Effect
	Alone	Combined		
NC34	20	10	1.0	Indifferent
INH	0.23	0.12		
NC34	20	20	2.0	
INH	0.23	0.23		
NC34	10	5	1.0	
MOX	0.2	0.1		
NC34	20	10	1.0	
MOX	0.2	0.1		
NC34	20	40	2.5	
ETH	16	8		
NC34	20	20	2	
ETH	8	8		
NC34	20	2.5	0.38	Synergism
RIF	0.015	0.0038		
NC34	20	5	0.38	
RIF	0.03	0.0038		

*^a^* Two-three independent experiments were performed. *^b^* Results of FICI below 0.5 indicate a synergistic outcome, from 0.5 to 4.0 suggest that each compound acts independently (indifferent), and above 4.0 indicates an antagonistic combination [17]. INH, isoniazid; MOX, moxifloxacin; ETH, ethambutol; RIF, rifampicin.

**Table 3:** Binding energy values of the compounds under study according to the values of the MolDock Score algorithm.

ID	DprE1 (PDB: 4FDO)	Dihydrofolate Reductase (PDB: 5JA3)
1. NC30	−73.214	−81.3373
2. NC31	−81.121	−89.7561
3. NC34	−75.389	−95.183
4. NC40	−87.517	−97.4009
5. NC36	−75.999	−89.989
6. NC53	−74.905	−89.7228
7. NC41	−86.800	−89.6239
8. NC51	**−95.1936 #**	−99.7266
9. Lig PDB	−86.972	**−155.333 #**

**#** The compound with the lowest energy is in bold.

## Data Availability

The data used to support the findings of this study are available from the corresponding authors upon request.

## Supplementary Materials

The following supporting information can be downloaded at: https://www.mdpi.com/article/10.3390/microorganisms13020396/s1, Table S1: Schematic of the two-drug combination test and concentrations (in µM) used for NC34 (in red) plus INH (isoniazid, in blue); Table S2: Schematic of the two-drug combination test and concentrations (in µM) used for NC34 (in red) plus RIF (rifampicin, in purple); Table S3: Schematic of the two-drug combination test and concentrations (in µM) used for NC34 (in red) plus ETH (ethambutol, in green); Table S4: Schematic of the two-drug combination test and concentrations (in µM) used for NC34 (in red) plus MOX (moxifloxacin, in pink).
