# Supplementary material for: Preclinical Evaluation of Selene-Ethylenelacticamides in Tuberculosis: Effects Against Active, Dormant, and Resistant Mycobacterium Tuberculosis and In Vitro Toxicity Investigation
Source: Microorganisms. 2025 Feb 11;13(2):396. doi: 10.3390/microorganisms13020396 (PMC11858155; doi:10.3390/microorganisms13020396)
Supplement: Supplementary file 1 [file microorganisms-13-00396-s001.zip › microorganisms-3454618-supplementary.pdf]

Supplementary Material

Table S1: Schematic of the two-drug combination test and concentrations (in µM) used for NC34 (in red) plus INH (isoniazid, in blue).

**NC34** + **INH**	1	2	3	4	5	6
A	20 + 0.94	20 + 0.47	20 + 0.23	20 + 0.12	20 + 0.06	20 + 0
B	10 + 0.94	10 + 0.47	10 + 0.23	10 + 0.12	10 + 0.06	10 + 0
C	5 + 0.94	5 + 0.47	5 + 0.23	5 + 0.12	5 + 0.06	5 + 0
D	2.5 + 0.94	2.5 + 0.47	2.5 + 0.23	2.5 + 0.12	2.5 + 0.06	2.5 + 0
E	1.25 + 0.94	1.25 + 0.47	1.25 + 0.23	1.25 + 0.12	1.25 + 0.06	1.25 + 0
F	0 + 0.94	0 + 0.47	0 + 0.23	0 + 0.12	0 + 0.06	0 + 0

Table S2: Schematic of the two-drug combination test and concentrations (in µM) used for NC34 (in red) plus RIF (rifampicin, in purple).

**NC34** + **RIF**	1	2	3	4	5	6
A	20 + 0.06	20 + 0.03	20 + 0.015	20 + 0.075	20 + 0.004	20 + 0
B	10 + 0.06	10 + 0.03	10 + 0.015	10 + 0.075	10 + 0.004	10 + 0
C	5 + 0.06	5 + 0.03	5 + 0.015	5 + 0.075	5 + 0.004	5 + 0
D	2.5 + 0.06	2.5 + 0.03	2.5 + 0.015	2.5 + 0.075	2.5 + 0.004	2.5 + 0
E	1.25 + 0.06	1.25 + 0.03	1.25 + 0.015	1.25 + 0.075	1.25 +0.004	1.25 + 0
F	0 + 0.06	0 + 0.03	0 + 0.015	0 + 0.075	0 + 0.004	0 + 0

Table S3: Schematic of the two-drug combination test and concentrations (in µM) used for NC34 (in red) plus ETH (ethambutol, in green).

NC34 + ETH	1	2	3	4	5	6
A	20 + 16	20 + 8	20 + 4	20 + 2	20 + 1	20 + 0
B	10 + 16	10 + 8	10 + 4	10 + 2	10 + 1	10 + 0
C	5 + 16	5 + 8	5 + 4	5 + 2	5 + 1	5 + 0
D	2.5 + 16	2.5 + 8	2.5 + 4	2.5 + 2	2.5 + 1	2.5 + 0
E	1.25 + 16	1.25 + 8	1.25 + 4	1.25 + 2	1.25 + 1	1.25 + 0
F	0 + 16	0 + 8	0 + 4	0 + 2	0 + 1	0 + 0

Table S4: Schematic of the two-drug combination test and concentrations (in µM) used for NC34 (in red) plus MOX (moxifloxacin, in pink).

NC34 + MOX	1	2	3	4	5	6
A	20 + 0.4	20 + 0.2	20 + 0.1	20 + 0.05	20 + 0.025	20 + 0
B	10 + 0.4	10 + 0.2	10 + 0.1	10 + 0.05	10 + 0.025	10 + 0
C	5 + 0.4	5 + 0.2	5 + 0.1	5 + 0.05	5 + 0.025	5 + 0
D	2.5 + 0.4	2.5 + 0.2	2.5 + 0.1	2.5 + 0.05	2.5 + 0.025	2.5 + 0
E	1.25 + 0.4	1.25 + 0.2	1.25 + 0.1	1.25 + 0.05	1.25 + 0.025	1.25 + 0
F	0 + 0.4	0 + 0.2	0 + 0.1	0 + 0.05	0 + 0.025	0 + 0
